# ‘I’m proud of my son with CP’: Cerebral palsy caregivers’ experiences, Gauteng province

**DOI:** 10.4102/ajod.v13i0.1357

**Published:** 2024-06-27

**Authors:** Faith Maronga-Feshete, Sonti Pilusa, Abigail Dreyer

**Affiliations:** 1 Department of Rural Health, Faculty of Health Sciences, University of the Witwatersrand, Johannesburg, South Africa; 2 Department of Physiotherapy, Faculty of Health Sciences, University of the Witwatersrand, Johannesburg, South Africa

**Keywords:** cerebral palsy, caregivers, challenges, negative experiences, positive experiences

## Abstract

**Background:**

Caregivers of children with cerebral palsy (CP) are critical in the survival and well-being of their children. Despite the caregivers’ particularly demanding responsibilities, literature on their experiences is limited.

**Objectives:**

This study explored the caregivers’ experiences of providing care to children with CP.

**Method:**

An explorative qualitative study design using semi-structured interviews was employed. All interviews were audio-recorded, transcribed verbatim and analysed guided by Colaizzi’s seven-step methodology.

**Results:**

Two themes emerged: the challenges in caregiving and positive experiences of providing care. Caregivers faced financial, psychological, social and physical challenges such as stigmatisation, a lack of work accommodations, time constraints due to demands of providing care, strained family relations, isolation, exclusion, emotional and physical exhaustion in their caregiving role. Despite the challenges, they also had fulfilling, positive experiences. Caregivers became more resilient, some relationships were strengthened and awareness of the CP condition increased over time.

**Conclusion:**

Caring for a child with CP is challenging. Cerebral palsy is a permanent disability; therefore, a holistic, long-term perspective to supporting caregivers is necessary to ensure they can care for their children adequately.

**Contribution:**

There is a need for various support structures for caregivers to lessen the burden of care. It is necessary to establish the relationships between the support structures available and the way that these structures are viewed and consequently utilised by the caregivers. This study highlights the experiences and needs of caregivers to inform stakeholders on intervention strategies.

## Introduction

Cerebral palsy (CP), a neurological nonprogressive impairment, is reported to be the most common cause of childhood disability globally, with long-term physical and social consequences (Zuurmond et al. 2018). The global prevalence of CP has been estimated between 2.0 and 2.5 cases per 1000 live births. However, it is higher in low-income areas. Cerebral palsy is more prevalent in poorly resourced settings such as countries in sub-Saharan Africa (Mangamba et al. 2022). In African settings, CP prevalence was estimated at up to 10 cases per 1000 births (Malla et al. 2022). Even this high record of cases may still be an underestimate because of low reporting of neurologic conditions in the African context (Malla et al. 2022). In South Africa, the prevalence of CP has also been estimated at 10 cases per 1000 live births (Zuurmond et al. 2018).

Children with CP may have sensory, physical and intellectual deficits that limit mobility and self-care activities, such as independent feeding, dressing and bathing (Bearden et al. 2016). Sensation, cognition, perception, musculoskeletal development, muscle tone, movement, posture, communication and behaviour may be impaired (Morgan & McGinley 2018). Other comorbidities include visual, speech and hearing impairments, orthopaedic deformities, general poor health, malnutrition because of feeding problems and epilepsy (Gonzalez et al. 2023; Malla et al. 2022). Due to the chronic nature of CP and its multiple comorbidities, CP has a significant impact on the quality of life (QOL) of the child and their caregiver (Fairfax et al. 2019). For the child, the multiplicities of health challenges imply a lifetime of dependency. For the caregiver, it implies long-term, constant provision of care (Kvarme et al. 2016).

Care needs for children with CP may exceed the expectations of the caregivers. In most cases, caregivers are not adequately prepared to execute this demanding role, resulting in significant strain on the caregiver (Chiluba & Moyo 2017). While some families may be able to negotiate this path, others require assistance as they face the medical, psycho-social and financial strains of having a member with CP (Sayed, Alaskar & Alonazi 2020). Caregivers providing such informal care are also likely to experience negative health outcomes because of the emotional and physical impact of care, social and cultural factors, financial demands and demands for providing care for other family members (Sayed et al. 2020; Dambi et al. 2016). One study done in Zambia showed high scores of long-standing strain and high psychiatric morbidity in caregivers of children with CP (Chiluba & Moyo 2017). Another study done on Polish primary caregivers revealed that caregivers of children with CP experience higher levels of anxiety and depression compared to caregivers of normally developing children (Gugała et al. 2019). This is also supported by a Kenyan study, which indicated that not only are caregivers of children with CP more stressed, but their households are also poorer (Hunt et al. 2021).

It is therefore important to ask what the experiences of caregivers of children with CP are regardless of their economic standing, social or cultural contexts. This study explored the experiences of South African caregivers of children with CP and presented the challenges, struggles and achievements in their role. This study will be beneficial in informing and directing intervention efforts in lessening the burden of providing care.

## Research methods and design

### Study design

An explorative qualitative design was used to give an in-depth understanding of the experiences of primary caregivers. Semi-structured interviews were conducted to explore and capture personal experiences of participants. The study design was appropriate to understand complex life experiences and extract the unique experiences of caregivers. The use of techniques such as probing, observations in the caregiver’s home environment, note-taking during the interview all combined to give depth and detail to the interview.

### Study setting

The study was conducted in Diepsloot, a predominantly informal settlement in Johannesburg, Gauteng province, South Africa. Diepsloot is in Region A of Johannesburg Metropolitan Municipality, 40 km north of the City of Johannesburg (Mahajan 2014). The township of Diepsloot emerged as people were being evicted from neighbouring farms and privately owned land in the 1990s. Currently, Diepsloot’s population is estimated at over half a million (Cahill 2019). In addition to the migration of South Africans to Diepsloot, the area has also received many unregistered immigrants who come as job seekers from other African countries. High unemployment rates and crime levels, poor water and sanitation systems, issues of adequate and appropriate housing, service delivery and effective local governance plague Diepsloot. The existing amenities are overburdened by the increasing population (Mahajan 2014). Most families are dependent on government social grants. The most accessed grants include the child support grant, old persons’ grant and the disability grant. Though these financial assistance platforms are available, beneficiaries are limited to South African nationals, permanent residents and registered refugees (Mahajan 2014).

### Study population

The study population comprised of primary caregivers of children with CP whose children attended a daycare centre at an NPO for people living with disabilities in the area. The centre allowed the researcher to send letters to families *via* the children at the end of their school day. The letter given to the children briefed families of the intended study. Caregivers responded through a tear-off slip on the letter, that they would be willing to participate. They provided their contact details that the researcher used to contact them telephonically and establish who the primary caregivers were.

A primary caregiver was defined as the individual who was mostly responsible for taking care of the child at home. To meet the inclusion criteria, the primary caregiver and the child had to reside in Diepsloot or immediate surroundings, the child under their care must have been medically diagnosed with CP and under the age of 18. There was a total of 10 primary caregivers whose children attended the daycare centre who were eligible for the study. All 10 caregivers consented to participating and were all recruited into the study.

### Data collection

Section A of the interview guide captured the demographic information of the child (age, gender, diagnosis, gross motor functional level) and that of the primary caregiver (age, gender, level of education and employment status). Section B consisted of open-ended questions that targeted the caregivers’ experiences in providing care. A pilot study was conducted in which two caregivers were interviewed to ascertain the adequacy of the research tool and to be acquainted with the process of data collection. The interviews were conducted in participant’s preferred language. Field notes were taken from the researcher’s observations of body language, family interactions and living conditions. This was relevant in providing more detail about the conditions within which families lived and observe any struggles or challenges that existed but may not be reported. Observing expressions in facial or body language provided an opportunity for the researcher to probe further when necessary and add depth to the information obtained during the interview. All the interviews were recorded using an audio-recorder. The coronavirus disease 2019 (COVID-19) protocols were observed in all interviews. Data collection commenced from January 2021 to April 2021.

### Data analysis

The audio interviews were transcribed verbatim in the language that the interviews were conducted by an independent transcriber. Transcripts were then translated into English. Translations were checked before analysis by the first author. Interviews were exported to MAXQDA 11 version 18.2.0. The software was used in the general sorting and organisation of data as well as analysis. Data were analysed using thematic analysis, transforming text into meaningful units, codes, categories and themes. The authors individually coded a transcript inductively, followed by a discussion on the codes and the categories. A coding framework was developed and used to code the rest of the transcripts. Categorisation was conducted by the first author and reviewed by the co-authors. A further abstraction of the data to develop themes related to the study objective was performed by all the authors. Below is a summary of the steps taken in the data analysis.

### Summary of the Colaizzi’s thematic analysis process methodology (Nowell et al. 2017)

1. Familiarisation with the data was done through re-reading the scripts to gain understanding of the content.
2. Generation of initial codes was done through identifying and labelling codes.
3. Theme searching was done through searching the data for recurring points to obtain the themes in the codes identified in step 2.
4. Reviewing and refining themes done through refining the identified themes.
5. Naming themes through clearly listing themes and giving clear descriptions of themes.
6. Writing the analysis where researchers compiled the report, presenting the findings of the analysis.

Member checking was done telephonically on some of the interviews. The author established dependability of the study through detailing the study process in logical and easily traceable documentation. Through providing a detailed description of the study settings, the researcher ensured that transferability to a similar context may be applicable. An external translator was used to check for accuracy of translations and increase objectivity of the transcriptions. There was collaboration with peers and co-authors during coding.

### Ethical considerations

An application for full ethical approval was made to the University of Witwatersrand, Human Research Committee (Medical) and ethics consent was received on 04 December 2020. Ethical clearance to conduct this study was obtained from the University of Witwatersrand, Human Research Committee (No. M2011133).

## Results

Ten caregivers, eight mothers and two fathers of children with CP, were interviewed. The age range of caregivers was from 28 to 60 years of age.

The ages of the children whose caregivers were interviewed ranged from 4 to 17 years of age. Four were girls while six were boys. Only two children were functionally ambulant. One used a walking frame and the other self-propelled on the floor. At the time of the interviews, none of the children attended day-care or special needs schools because of the COVID-19 school restrictions. Table 3 illustrates the children’s age, GMFCS levels and the types of assistive devices used.

**TABLE 1:** Demographic information of caregivers.

Demographic variable	Subdivision	Number of caregivers
Gender	Male	2
	Female	8
Age	25–40	4
	41–55	3
	Over 55	3
Marital status	Married	7
	Divorced	2
	Widowed	1
Level of education	Below Grade 10	4
	Grade 11 to 12	4
	Diploma	2
Employment status	Unemployed	7
	Formally employed	3
Caregivers’ years of providing care	0–5 years	2
	6–10 years	2
	11–15 years	2
	16–20 years	4
Child support social grant	Yes	8
	No	2
Housing	Brick and mortar	10

**TABLE 2:** Demographic profile summary of the children in the study.

Demographic variable	Subdivision	Number
Age	Range from 4 to 17 years	Average 8.9 years
Sex	Male	6
	Female	4
GMFCS	5	8
	4	2
Assistive device	Shona buggy	4
	Baby stroller	2
	Walking frame	1
	Car seat and wheelchair	3

Note: GMFCS is a grading system for children with CP into levels 1 to 5 based on their gross motor function, level 1 being independently mobile and level 5 being severely limited mobility even with adapted assistive devices.

**TABLE 3:** GMFCS level and assistive devices.

Caregiver†	Sex	Age of child	GMFCS level	Assistive devices
Anna	M	4 years	5	Shona buggy
Busi	M	6 years	5	Shona buggy
Carol	F	4 years	4	Baby stroller
Dora	F	8 years	5	Baby stroller
Esther	F	16 years	4	Walking frame, wheelchair
Fiona	M	17 years	5	Shona buggy
Gilbert	M	16 years	5	Shona buggy
Hugo	M	15 years	5	Wheelchair
Isabel	M	15 years	5	Car seat, wheelchair
Joy	F	16 years	5	Car seat, Wheelchair

Note: GMFCS is a grading system for children with CP into levels 1 to 5 based on their gross motor function, level 1 being independently mobile and level 5 being severely limited mobility even with adapted assistive devices.

† , Pseudonym.

### Themes

Two themes emerged from the study, namely challenges in caregiving and positive experiences in caregiving.

## Challenges in caregiving

The caregivers expressed challenges they experience when providing care to the children with CP. The challenges affected the financial, emotional, physical and social aspects of their lives.

### Financial

Taking care of a child with CP was costly because of the additional special needs of a child with a disability such as diapers, dietary supplements and special food transport cost, hiring temporary care and paying fees in special schools. These needs require substantial finances:

‘It is very expensive actually to have a disabled kid because you need a lot of things … Pampers, then the kind of food you give him, you must make sure you choose the right food.’ (Gilbert, child 16 years, GMFCS 5)

‘You know when you buy her pampers, for example, a pack is R310.00 they don’t last her the whole month. You buy at least twice … What is she going to eat? You start to have stress … You also must have transport money to go to the hospital to fetch her medication. When you leave her with someone from outside you must pay.’ (Joy, child 16 years, GMFCS 5)

**FIGURE 1:**
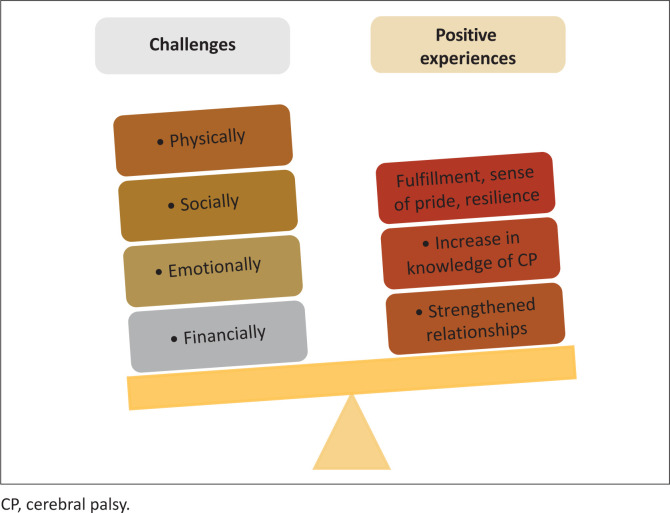
Themes and subthemes on experiences of caregivers of children with cerebral palsy.

### Social

Taking care of a child with CP was also socially challenging. Work, relationships and community perceptions on disability made caregiving difficult. Incorrect traditional beliefs about disability affected the caregivers’ ability to accept their children’s condition:

‘I avoid those ones. I don’t care what they say. I look after my child only, I know that they talk, but I don’t care … and as I was saying I am not used to a lot of people, I don’t even go to other people’s houses and there is no one who comes here.’ (Joy, child 16 years, GMFCS 5)

‘When he was still young the one other thing I hated was when someone came to me and said ‘shame’ to me. I hate that word, “shame”. Sometimes it’s even an older person, you can’t talk back, and they will say shame and then my tears will start rolling. At that time, you can see that when you come with him, people will start distancing themselves and then look at you like it is for the first time that they see something like that … So, I will get more angry. “Why can’t you ask me, why do you look at him like that.” I get frustrated.’ (Fiona, child 17 years, GMFCS 5)

‘If you have a baby and I see your baby and I don’t say “hello, hello baby”, to play with the baby, then what do you think? Can you say this person is happy for my child? Or sometimes they say, “hey boy you don’t talk.” They don’t say “hello boy”, they say, “oh you don’t talk”, then what am I supposed to think? It’s useless to talk to them, just keep quiet. The main thing with these kids is it’s not easy for other people to accept especially somebody who doesn’t have a kid like that in his or her family, it’s not easy.’ (Gilbert, child 16 years, GMFCS 5)

### Physical

Caregivers highlighted the physical strain they experienced when caring for their children with CP. As the children grew older, they became heavier and difficult to manage:

‘Now Tshepo is growing, many things I fail to do with him because even lifting him up hurts my back because he is heavy.’ (Hugo, child 15 years, GMFCS 5)

‘So, the biggest challenge I have is that he’s growing. We can’t put him on our backs anymore. When you carry him, he’s tall, and then when I carry him, he looks thin, but he is heavy, I don’t want to lie.’ (Fiona, child 17 years, GMFCS 5)

‘He is too heavy, but we have to lift him to the bath, and we sit him on that kids’ plastic chair and then bath him there.’ (Isabel, child 15 years, GMFCS 5)

As the children become older, the caregivers worried about their future and who would take over the care if anything happened to them. Joy expressed that:

‘There is no one who is helping me … It’s me alone, there’s no one even from my family. So, if I am no longer here what will happen?’ (Joy, child 16 years, GMFCS 5)

### Vocational challenges

Work life and work prospects were affected. Finding and keeping work was not easy for the caregivers, thus affecting their family financial status:

‘[*I*]t [*CP*] changed my life because I didn’t have time to look for a job. I was looking after her all the time until I found this job where I can come with her to work because it is a creche.’ (Carol, child 4 years, GMFCS 4)

Another caregiver reported:

‘… I must take care of him; I can’t go to a job. Who will look after him when I am not here?.’ (Hugo, child 15 years, GMFCS 5)

### Family relationships

Other social challenges included strained relationships. Some of the caregivers were abandoned by their partners:

‘As I said, at first, I told you how my son was, big hair, big eyes and he didn’t have a forehead and he was thin … I could see his father struggling, I never attended a support group at hospital, he is the one that was attending because he couldn’t accept the condition. That is when we broke up because I saw that he was blaming me a lot.’ (Fiona, child 17 years, GMFCS 5)

### Communication with the child

The caregivers struggled to relate with their children with CP. They expressed difficulty in understanding the needs of the children and they had to rely on their instincts. Inability to communicate with their children caused a lot of stress:

‘[*A*]nd sometimes we don’t know anything. I could be trying to feed him, and he doesn’t want, and I don’t know if he doesn’t want to be fed by me, or the food is not nice or the food isn’t right, what is wrong with the food, is it not enough sugar, is it not enough salt, what do you want? So, it’s a big challenge to feed. Feeding only, and there are other things that need to be done… If they can help us to make sure these kids can communicate. That is the main thing, communication. If he can try to communicate and say I want to do this, I want to do this., I know he cannot speak but sign language or whatever he can say yes or no.’ (Gilbert, child 16 years, GMFCS 5)

Another caregiver shared the same experience. She indicated that:

‘[*T*]he challenging thing is that he can’t talk, and everything you must think for him, like what do you want? Even if he is in pain, you can just see that something is wrong but where, you don’t know. You just must take him to the doctor because you can’t give him medication when you don’t know what is wrong.’ (Busi, child 6 years, GMFCS 5)

### Emotional challenges

Caregiving was emotionally challenging, impacting the well-being and self-care of caregivers. Some caregivers felt disappointment from not having a healthy child as expressed below:

‘At first, I was disappointed; I was not accepting this condition. I was stressed, losing weight because of stress. … it’s not an easy thing to cope with it but you must tell yourself this is what God gave me. Maybe with that we are trying to tell ourselves so that we cope but it’s frustrating.’ (Gilbert, child 16 years, GMFCS 5).

‘[*W*]as disturbed because, you know when you have had kids before who were never like this you start to have stress and asking yourself what has happened now, what is happening.’ (Esther, child 16 years, GMFCS 4)

Caregivers expressed how they overlooked their personal well-being because care-giving was time consuming and thus they neglected their self-care.

‘[*A*]t first I felt like I was running like I’m losing my mind, not knowing what to do because I was focusing more on him [*child with CP*] than on myself and I was always stressed … I focus on him a lot and so my own time is very little.’ (Fiona, child 17 years, GMFCS 5)

## Positive experiences in caregiving

Despite the challenging experiences, there were positive experiences of providing care. The whole experience of caregiving shaped caregivers’ outlook towards life. The caregivers’ relationships were strengthened as expressed:

‘Our marriage became more stable. Now we are also praying, and we are more open with each other and that helps us.’ (Dora, child 8 years, GMFCS 5)

‘I believe, luckily, we had so much special love for Gift [*child with CP*] so that made us think that Gift is not her [*the mother*] responsibility alone or my responsibility alone and that made our marriage survive.’ (Isabel, child 15 years, GMFCS 5)

For other caregivers, friendships and support structures were forged based on the common role of caregiving:

‘Yesterday I spoke to another caregiver of a child with CP… She asked how my son was doing, and I also asked how her child was doing? They came to my son’s birthday in October, November we went for her son’s birthday.’ (Fiona, child 17 years, GMFCS 5)

For other caregivers, the whole experience built their resilience as they learnt to accept their situation and developed a sense of confidence in their role. For example, Gilbert said:

‘I have accepted a lot, both my child’s situation and mine. It’s not easy. Because I have accepted, it looks like it’s easy and yet it’s still not easy … I think I’m fine. I have done this [*caregiving*] for the past 16 years and yoh, that is a long time. I think I’m fine now.’ (Gilbert, child 16 years, GMFCS 5)

In addition, the caregivers learnt a lot from taking care of their children with CP. Through different courses, they learnt about the CP condition and skills on how to care for a person with a disability:

‘Being a mother of a child with CP comes with a lot of challenges, but then you also learn a lot, a lot. You get more knowledge about health, especially CPs.’ (Busi, child 6 years, GMFCS 5)

‘Last year, my child wasn’t crawling. So, they taught me how to do the exercises to help her learn to crawl. I started going to the NGO for people with disability when she wasn’t sitting down. Now, she can sit, she can crawl, I’m looking forward to seeing my child stand or to walk.’ (Carol, child 4 years, GMFCS 4)

The caregivers learnt problem solving skills and could seek for more information when they needed it:

‘When I get home from hospital, I will remember something they told me, I will Google and find more information about what’s going on. At first, I didn’t know that he had quadriplegia because they didn’t tell me that he is a quadriplegic. I only heard the doctor saying it, after the students went out then I ask him what he meant when he said my child is a quadriplegic. I googled and got even more information.’ (Fiona, child 17 years, GMFCS 5)

Caregivers had a sense of pride and joy in their children despite their challenges:

‘I’m proud of my son. I’m proud and I’m happy with him.’ (Gilbert, child 16 years, GMFCS 5)

‘I want to tell them the way Tshepi is. He is friendly, he likes people, and he likes gospel music. He makes me happy.’ (Hugo, child 16 years, GMFCS 5)

## Discussion

### Challenging experience of providing care

Having a child with CP is traumatic, with feelings of loss, anger, shock and denial (Fernández-Alcántara et al. 2015). This study found that the caregivers experienced challenges when providing care to their children with CP, affecting their emotional, physical, financial and social lives.

Caregivers become prone to emotional stress, depression and sometimes repressed hostility (Singogo, Mweshi & Rhoda 2015; Vadivelan et al. 2020). One study done in Zimbabwe highlighted that CP was associated with evil, viewed as punishment from ancestors for the mother’s wrongdoing (Muderedzi et al. 2017). In Kenya, mothers of children with CP reported feelings of stigma and shame, with their children being treated as if they were not human (Bunning et al. 2017). Similar to other studies, this study showed that some mothers were abandoned by their spouses, and some family members were unwilling to care for or even touch the child. A lack of knowledge and public awareness of the causes of CP may cause such discrimination and stigmatisation, leading to social withdrawal of caregivers and their children.

Financial constraint is a common barrier to providing optimum care. This is because of extra costs in transportation, therapies, assistive devices, special diets, nappies and the lack of appropriate beneficial economic opportunities for caregivers. Without the financial capacity to meet basic needs, the role of caregiving is distressful, in many cases forcing caregivers to forego their own needs (Trindade et al. 2020). Financial costs associated with caring for a child with CP may affect a family’s financial stability (Hunt et al. 2021). Lack of work accommodations, time constraints and caregiver-fatigue and high rates of hospitalisations predispose families to an increased risk of poverty (Carter et al. 2021; Simeu & Mitra 2019). Transport and hospital-stay costs alone place a major financial strain on caregivers and their families. Social integration of the child may become unaffordable (Paajanen, Annerstedt & Atkins 2021). These factors may contribute to increased stress and somatic illnesses as well as perpetuating the cycle of poverty, hence family donations or governmental social grants become critical (Gamarra et al. 2020).

Caregivers invest their effort, time and finances, sometimes at the expense of their emotional well-being and physical health, careers and other family members. The QOL of the caregiver and the family as a whole may be negatively affected (Trindade et al. 2020). Other common challenges are strained marital relations, family and community stigmatisation, physical and emotional exhaustion, isolation of the child and social exclusion of the caregiver (Menlah & Osei 2020). Traditional or social beliefs shape attitudes and affect the parenting, caregiving capabilities, psychological and physical health of caregivers (Muderedzi et al. 2017; Donald et al. 2014). As a result of stigmatisation, caregivers do not seek support or engagement in relations that may be helpful, forcing them to isolate and carry the burden of caregiving on their own (Wijesinghe et al. 2015).

In addition to these social difficulties, the uncertainty in caregiving skills required when handling complications of CP makes the caregiving role even more daunting. A lack of information (or the poor communication of it) and the lack of continued long-term care from healthcare professionals compound the challenges of providing care, leaving caregivers feeling alone and unsupported (Nuri, Aldersey & Ghahari 2019). The lack of appropriate assistive devices is a major barrier faced by most caregivers in resources-strained settings and it worsens the challenges of providing care (Hanass-Hancock et al. 2017). Caregivers in resource-deprived settings carry their children on their backs when attending medical appointments on account of a lack of assistive devices, the appropriateness of assistive devices or difficult terrain (Donald et al. 2014).

### Positive experiences

#### Resilience and fulfilment

Not all experiences of caregivers are negative. Caregivers also have rewarding and fulfilling outcomes in performing their caregiving roles (McKenna et al. 2022). Being a caregiver of a child with disabilities in a resource-strained environment resulted in building resilience and fortitude over time (McKenna et al. 2022). Even though all caregivers in the current study had challenges, they resiliently carried on with their roles, finding fulfilment in their children and in caring for them. They managed to continuously shift and adapt to the physical, psychological and socio-economic demands of the caregiving role. Resilience is dynamic and not necessarily an inherent state that is immovable (McKenna et al. 2022). As such, resilience can be built and can fluctuate through different phases of challenges. The quality of being resilient cushions caregivers from negative outcomes such as burnout and frustration resulting in relationship satisfaction, positive affect and better QOL (McKenna et al. 2022). Through built resilience and despite the burden of care, caregivers were able to experience joy and pride from their children. Affection from care recipients brings fulfilment to the caregiver (Lin, Rong & Lee 2013). Caregivers receiving affection and gratitude from their children with CP in gestures such as smiling, recognition of voices and showing excitement or contentment brings fulfilment to the caregiver.

### Increase in the knowledge and management of cerebral palsy

Pushed by the uncertainty of their competence to perform their role or even fear of doing something wrong in the caregiving role, caregivers actively engaged in acquiring information from different sources (Huang, Kellett & St John 2010). They acquired skills and information from therapists, the internet and from each other. Such knowledge was helpful in alleviating the stress and burden of caregiving.

### Strengthened relationships

The common thread of findings indicates that having a child with a disability negatively impacts relationships (Mitra et al. 2017). On the contrary, this current study revealed that some marriages were strengthened over the common goal of protecting and providing for the child with CP. In support of the study’s findings, strengthened family relationships as an outcome of providing care has been noted in long-term neurological conditions in other settings (McKenna et al. 2022).

## Conclusion

The experience of caregiving has both challenging and positive experiences. Caregivers of children with CP are faced by a myriad of complex challenges that require multi-dimensional interventions. Issues of poverty, discrimination, compromised access to health care and the lack of knowledge compromise the health and well-being of caregivers and, inadvertently, that of the children in their care. The interconnectedness of these challenges requires a comprehensive approach but also indicates that a single intervention may have a ripple effect into other areas of caregiving.
